# Allergic diseases of the skin and drug allergies – 2032. Epidemiological study of a specific unit of drug allergy diagnosis - 20 years experience

**DOI:** 10.1186/1939-4551-6-S1-P118

**Published:** 2013-04-23

**Authors:** Enrique Marti, Enrique Scorza

**Affiliations:** 1 Hospital Sant Joan Despi, Moisés Broggi., Barcelona, Spain

## Background

To determine the allergy incidence to drugs in patients visited in our unit with a clinical history suggestive of allergic hypersensitivity to one or more drugs. Our area of influence is the whole region of Catalonia, North-East of Spain.

## Methods

We use our own method of diagnosis; keeping in mind the limitations in our medical practice such as a difficult anamnesis; few standardised skin tests for most substances; and the scarce reliability of the in-Vitro tests. The provocation test was the gold standard of the definitive diagnosis.

## Results

33750 patients have been studied in 20 years; each patient has undergone an average of 1.4 tests 68% of them being female and 32% male.

## Conclusions

A positive result was found in 20% of the studied cases; 8% of these related to placebo. The most studied drugs have been the beta lactamates (50%), non-steroid anti-inflamatories (NSAIDs) (30%), local anesthesics (10%), others (10%).

